# Genomic Signatures of Human versus Avian Influenza A Viruses

**DOI:** 10.3201/eid1209.060276

**Published:** 2006-09

**Authors:** Guang-Wu Chen, Shih-Cheng Chang, Chee-Keng Mok, Yu-Luan Lo, Yu-Nong Kung, Ji-Hung Huang, Yun-Han Shih, Ji-Yi Wang, Chiayn Chiang, Chi-Jene Chen, Shin-Ru Shih

**Affiliations:** * Chang Gung University, Taoyuan, Taiwan, Republic of China

**Keywords:** human influenza, avian influenza, host specificity, genome, sequence analysis, research

## Abstract

Position-specific entropy profiles created from scanning 306 human and 95 avian influenza A viral genomes showed that 228 of 4,591 amino acid residues yielded significant differences between these 2 viruses. We subsequently used 15,785 protein sequences from the National Center for Biotechnology Information (NCBI) to assess the robustness of these signatures and obtained 52 "species-associated" positions. Specific mutations on those points may enable an avian influenza virus to become a human virus. Many of these signatures are found in NP, PA, and PB2 genes (viral ribonucleoproteins [RNPs]) and are mostly located in the functional domains related to RNP-RNP interactions that are important for viral replication. Upon inspecting 21 human-isolated avian influenza viral genomes from NCBI, we found 19 that exhibited >1 species-associated residue changes; 7 of them contained >2 substitutions. Histograms based on pairwise sequence comparison showed that NP disjointed most between human and avian influenza viruses, followed by PA and PB2.

## Abstract 2

Fifty-two species-associated amino acid residues were found between human and avian influenza viruses.

Pandemic influenza A virus infections have occurred 3 times during the past century; the 1957 (H2N2) and 1968 (H3N2) pandemic strains emerged from a reassortment of human and avian viruses (*1*). Recently, all 8 genome segments from the 1918 (H1N1) influenza A virus were completely sequenced. The results indicate that the 1918 pandemic virus may not have emerged by a reassortment of avian and human virus as did the 2 other pandemic strains. Although the 1918 H1N1 is not considered an avian virus, it is the most avianlike of all mammalian influenza viruses (*2**,**3*). The recent circulation of highly pathogenic avian H5N1 viruses in Asia from 2003 to 2006 has caused >90 human deaths and has raised concern about a new pandemic (*4*). Therefore, we need to understand what genetic variations could render avian influenza virus capable of becoming a pandemic strain. Genomewide comparison of human versus avian influenza A viruses would show the evolutionary similarities and differences between them and thus provide information for studying the mechanism of influenza viral infection and replication in different host species.

Although many research efforts have focused on the molecular evolution of specific genes of influenza viruses, comprehensive comparisons among the nucleotide sequences of all 8 genomic segments and among the 11 encoded protein sequences have not been extensively reported. In this study, we used several computational approaches for finding specific genetic signatures characteristic of human and avian influenza A viral genomes. We subsequently validated the robustness of those signatures with human and avian protein sequences downloaded from Influenza Virus Resources at the National Center for Biotechnology Information (NCBI) (http://www.ncbi.nlm.nih.gov/genomes/FLU/FLU.html).

## Materials and Methods

### Clinical Isolates

Throat swabs from patients with influenzalike syndromes were collected from the Clinical Virology Laboratory, Chang Gung Memorial Hospital. The specimens were inoculated in MDCK cells. Typing for influenza A virus was then performed with immunofluorescent assay by type-specific monoclonal antibody (Dako, Cambridgeshire, UK). Subtyping was conducted by reverse transcription (RT)–PCR with subtype-specific primers.

### Sequence Analysis

The RT-PCR product was purified by using the QIAquick Gel Extraction Kit (Qiagen, Valencia, CA, USA). The nucleotide sequence was determined with an automated DNA sequencer. Sequence editing and processing were performed with Lasergene, version 3.18 (DNASTAR, Madison, WI, USA). Multiple sequence alignment was performed with ClustalW version 1.83 (ftp://ftp.ebi.ac.uk/pub/software/unix/clustalw). Global sequence comparison that yielded pairwise sequence identities used in histogram analysis was done with the program Needle in the EMBOSS package (*5*). Amino acid sequences were translated from coding sequences and aligned by BioEdit (*6*). An entropy value was defined at an aligned amino acid position according to the formula ΣP_i_*log(*P_i_*), in which *i* is the observed probability for each of the 20 amino acids (aa) (*7*). A graphic tool was developed in Java for displaying the entropy plot used in this work. All amino acid numberings are based on influenza virus A/Puerto Rico/8/1934 (PR8).

### Sequences Used in Study

To show the host-associated amino acid signatures, we retrieved full genome sequences (as of August 22, 2005) from the genome browser at Influenza Sequence Database (ISD) (*8*). To differentiate between avian and human influenza viruses, we excluded human-isolated avian influenza viruses from the human dataset and examined those sequences separately. Altogether, we had 95 avian and 306 human influenza viral genomes, henceforth termed "primary dataset." All 11 viral proteins encoded by the 8 genomic RNA segments were compared: PB2, PB1, PB1-F2, PA, HA, NP, NA, M1, M2, NS1, and NS2.

Avian influenza viruses from human influenza patients were separately retrieved from NCBI as well as from ISD. Altogether, we had 417 protein sequences from 60 avian influenza strains, in which 21 strains contain sequences (full or nearly full length) from all 8 genomic RNA segments.

For validating the signatures obtained from analyzing the primary dataset, we further retrieved 15,785 human or avian influenza A viral protein sequences from NCBI's Influenza Virus Resources. Details for the sequences used can be found in Appendix, Supporting Materials and Methods, as well as in Table A1 and Table A2. Eleven Taiwanese genomes produced in this work have been deposited in GenBank with accession numbers DQ415283 through DQ415370.

## Results

### Differing Amino Acid Residues

**Figure A1:**
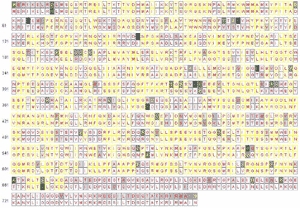
Entropy plot for all 11 influenza proteins for human (top) versus avian (bottom). In each aligned position, we have a consensus residue for 95 avian strains displayed on top, and a consensus residue for 306 human strains at the bottom. Completely conserved amino acid positions are filled with white, while less conserved amino acids are filled in various gray shadings. Positions where one single residue dominates over 90%, less than 90% but greater than 75%, and less than 75% are labeled with red, yellow, and green letters, respectively. Yellow rectangles indicate that both human and avian flu are completely conserved to the same residue, while rectangles in magenta indicate that avian and human flu each completely conserves to a different residue Additional plots for HA, NA, NS1 and NS2, for using different counts of human or avian strains are detailed as individual captions to these plots. Adobe Acrobat PDF available at http://wwwnc.cdc.gov/eid/pdfs/06-0276-FA1.pdf (21 pages).

Using previously described methods (*7*), we separately calculated an entropy value for every aligned amino acid position for 95 avian influenza viruses and 306 human influenza viruses. Those amino acid residues with an entropy value between 0 and –0.4 for both the human and avian strains were identified as most highly conserved. We chose this entropy threshold on the basis of the entropy value –0.379, calculated at position 627 of PB2 for the 95 avian viruses. This widely reported, species-associated residue is highly conserved; it has E (Glu) in 83 and K (Lys) in 12 avian isolates and Lys in all 306 human isolates. We then selected those conserved positions with distinct amino acid residues between human and avian influenza viruses as potential host-associated signatures. An entropy plot for identifying such signature residues for avian versus human influenza virus NP segments is shown in Figure panel A. In each aligned position, we placed an avian consensus residue on top and a human consensus at the bottom. For example, the entropy value is zero at amino acid position 283 for both avian and human strains, in which all 95 avian influenza viruses contain L (Leu), whereas all 306 human influenza viruses contain P (Pro). The other 2 residues with zero entropy value in avian and human viruses are located at position 55 of PA, in which we have D (Asp) in avian viruses and N (Asn) in human viruses, and position 121 of M1, in which we have T (Thr) in avian and A (Ala) in human viruses. Entropy plots for all 11 influenza viral proteins can be found in Figure A1.

Figure panel B shows a genomewide view of the entropy plots for 11 influenza A viral proteins. The amino acid sequences of hemagglutinin (HA), with an average entropy value of –0.524 within avian viruses and –0.158 within human viruses, exhibit much more diversity than other open reading frames (ORFs). PB2, PB1, PA, NP, and M1, on the other hand, are more conserved (i.e., they have less negative entropy values).

**Figure:**
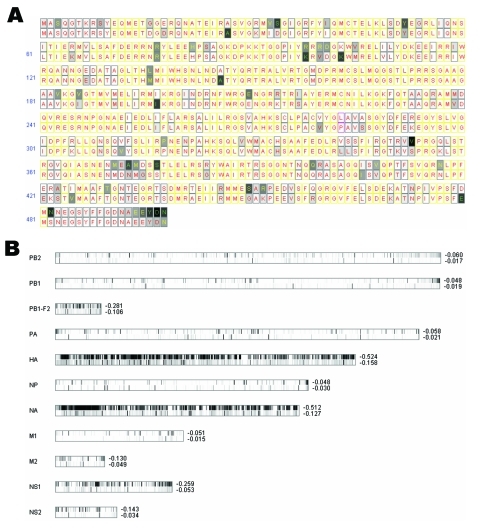
A) Entropy plot for avian versus human influenza viruses for NP amino acid residues. In each aligned position, we have a consensus residue for 95 avian strains displayed on top and a consensus residue for 306 human strains at the bottom. Completely conserved amino acid positions are filled with white; less conserved amino acids are filled in various gray shadings. Positions in which 1 single residue dominates >90%, <90% but >75%, and <75% are labeled with red, yellow, and green letters, respectively. Yellow rectangles indicate that both human and avian viruses are completely conserved to the same residue; magenta rectangles indicate that avian and human viruses are each completely conserved to a different residue. B) Entropy plots for the entire influenza A viral genome. Each lane displays entropy value distributions of aligned protein sequences for 1 of the 11 viral proteins; the upper half represents 95 avian strains, and the bottom half represents 306 human strains. (PB1-F2 contains fewer strains, as described in Discussion.) Positions completely conserved to a single residue are shown in a white band, while less conserved ones are shown in various gray shadings. The average entropy for the entire segment is shown to the right of these lanes. Entropy values are zero when residues are completely conserved; more negative values indicate more diversity. Alignment size for each protein from top to bottom is 759, 757, 90, 716, 591, 498, 480, 252, 97, 230, and 121.

In addition to the previously mentioned 3 positions with distinct amino acid residues between avian and human strains, we found 225 additional positions with nearly distinct amino acid residues, with their computed entropy values less negative than –0.4 in both the 306 human and 95 avian strains that we analyzed. To assess the robustness of those 228 residues used in differentiating human from avian influenza viruses, we further examined 15,785 influenza A protein sequences from NCBI. After validation, 52 positions still showed an entropy value less negative than –0.4 and conserved to distinct amino acid residues between human and avian viruses (Table 1). From this entropy analysis, we identified an additional 51 aa positions that may be as important as the well-known position 627 of PB2. We designated these 52 positions as "species-associated" signatures. Among 11 ORFs, NP contains the highest number of such signatures (15 positions), followed by PA (10 positions), PB2 (8 positions), PB1-F2 (5 positions), M2 (4 positions), M1 (3 positions), PB1 (2 positions), HA (2 positions), NS2 (2 positions), and NS1 (1 position). No signature was found in the NA gene. We also summarized the related functions of those species-associated signatures in Table 1. The complete results of genome scanning and validation can be found in Table A3 and Table A4.

**Table 1:** Validated amino acid signatures separating avian influenza viruses from human influenza viruses*

Gene	Position	Avian residues	Human residues	Associated functional domains
PB2	44	**A**(208),S(7)	**S**(831),A(10),L(2)	PB1–1, NP-1 (*9*), MLS (*10*)
	199	**A**(210),S(5)	**S**(842),A(3)	NP-1 (*9*)
	271	**T**(210),A(3),I(1),M(1)	**A**(836),T(6),S(1)	Cap-N (*11*)
	475	**L**(214),M(1)	**M**(839),L(3)	NLS (*12*)
	588	**A**(203),T(6),V(6)	**I**(835),V(3),A(2)	PB1–2, NP-2 (*9*)
	613	**V**(212),A(3)	**T**(816),I(16),A(8),V(1)	PB1–2, NP-2 (*9*)
	627	**E**(196),K(19)	**K**(838),R(2),E(1)	PB1–2, NP-2 (*9*)
	674	**A**(204),S(6),T(2),G(2),E(1)	**T**(836),A(2),I(2),P(1)	PB1–2, NP-2 (*9*)
PB1	327	**R**(147),K(3)	**K**(766),R(66)	cRNA (*13*)
	336	**V**(142),I(8)	**I**(773),V(59)	cRNA (*13*)
PB1-F2	73	**K**(397),R(6),I(1)	**R**(594),K(87),S(1)	ANT3, VDAC1 (*14*), mitochondrial localization (*15*), predicted amphipathic helix (*16*)
	76	**V**(401),A(3)	**A**(625),V(57)	ANT3, VADC1 (*14*), predicted amphipathic helix (*16*)
	79	**R**(369),Q(34),L(1)	**Q**(607),R(75)	ANT3, VADC1 (*14*), predicted amphipathic helix (*16*)
	82	**L**(382),S(22)	**S**(596),L(86)	ANT3, VADC1 (*14*), predicted amphipathic helix (*16*)
	87	**E**(389),G(14),K(1)	**G**(637),E(45)	ANT3, VADC1 (*14*)
PA	28	**P**(213),S(1)	**L**(831),P(9),R(2)	Proteolysis (*17*)
	55	**D**(214)	**N**(836),D(5)	Proteolysis (*17*)
	57	**R**(210),Q(4)	**Q**(829),R(6),L(4),K(2)	Proteolysis (*17*)
	225	**S**(213),C(1)	**C**(829),S(10)	Proteolysis (*17*), NLSII (*18*)
	268	**L**(214)	**I**(827),L(11), P(1)	
	356	**K**(212),X(1),R(1)	**R**(827),K(11)	
	382	**E**(208),D(5),V(1)	**D**(824),E(11),V(2),N(1)	
	404	**A**(214)	**S**(828),A(9),P(1)	
	409	**S**(189),N(24),I(1)	**N**(830),S(7),I(1)	
	552	**T**(213),N(1)	**S**(835),T(1),I(1)	
HA	237	**N**(582),R(49),D(2),H(1),S(1)	**R**(1209),N(12),S(2),D(1),K(1)	
	389	**D**(659),N(20),G(1),Y(1)	**N**(819),D(121)	
NP	16	**G**(356),S(9),D(6),T(2)	**D**(646),G(7)	RNA binding (*19*), BAT1/UAP56 (*20*), MxA (*21*), PB2–1 (*22*)
	33	**V**(355),I(18)	**I**(638),V(15)	RNA binding (*19*), MxA (*21*), PB2–1 (*22*)
	61	**I**(366),M(6),V(1)	**L**(642),I(8)	RNA binding (*19*), MxA (*21*), PB2–1 (*22*)
	100	**R**(360),K(11),V(2)	**V**(619),I(32),A(1),M(1)	RNA binding (*19*), MxA (*21*), PB2–1 (*22*)
	109	**I**(359),V(10),M(2),T(2)	**V**(614),I(34),T(3),A(2)	RNA binding (*19*), MxA (*21*), PB2–1 (*22*)
	214	**R**(352),K(20),L(1)	**K**(640),R(10)	NLS (*23*), CRM1 (*24*), NP-1 (*25*)
	283	**L**(372),P(1)	**P**(643),L(7)	NP-1 (*25*), PB2–2 (*22*)
	293	**R**(371),K(2)	**K**(622),R(28)	NP-1 (*25*), PB2–2 (*22*)
	305	**R**(369),K(4)	**K**(636),R(14)	NP-1 (*25*), PB2–2 (*22*)
	313	**F**(371),I(1),L(1)	**Y**(642),F(8)	NP-1 (*25*), PB2–2 (*22*)
	357	**Q**(368),K(4),T(1)	**K**(644),R(8),Q(1)	NAS (*26*), NP-1 (*25*), PB2–3 (*22*)
	372	**E**(357),D(15),K(1)	**D**(630),E(23)	NAS (*26*), NP-2 (*25*), PB2–3 (*22*)
	422	**R**(373)	**K**(630),R(23)	CTL epitope (*27*), NP-2 (*25*), PB2–3 (*22*)
	442	**T**(372),A(1)	**A**(629),T(23),R(1)	NP-2 (*25*), PB2–3 (*22*)
	455	**D**(373)	**E**(630),D(22),T(1)	NP-2 (*25*), PB2–3 (*22*)
M1	115	**V**(856),I(2),L(1),G(1)	**I**(981),V(9)	
	121	**T**(840),A(19),P(1)	**A**(988),T(2)	
	137	**T**(859),A(1),P(1)	**A**(974),T(12)	
M2	11	**T**(434),I(11),S(2)	**I**(911),T(44)	Host restriction specificities (*28*), ectodomain (*29*)
	20	**S**(471),N(13)	**N**(926),S(29)	Host restriction specificities (*28*). ectodomain (*29*)
	57	**Y**(481),C(1),H(1)	**H**(913),Y(33),R(2),Q(1)	CRAC (*30*), endodomain (*29*)
	86	**V**(378)	**A**(924),V(10),T(4),D(1)	Endodomain (*29*)
NS1	227	**E**(692),G(9),K(1),S(1)	**R**(897),G(5),K(1),E(1)	
NS2	70	**S**(453),G(21),D(1)	**G**(903),S(2)	M1, NEP dimerization domain (*31*)
	107	**L**(468),S(2),F(1)	**F**(777),L(16),S(1)	M1, NEP dimerization domain (*31*)

*Numbers in parentheses in residue columns are the number of sequences yielding the specific amino acid residue; **bold** indicates dominant amino acid residue type.

### Amino Acid Signatures in Human Viruses

We examined how the amino acid sequences varied at those proposed signature positions for avian influenza viruses isolated from humans. At 9 of these 52 positions, residue changes were characteristic of human rather than avian viruses (Table 2). For example, 34 sequences (27 H5N1, 3 H9N2, and 4 H7N7) were available for inspection at position 199 of PB2 (data not shown). Aside from 10 sequences with gaps (sequences did not cover this position), 19 of the remaining 24 still have Ala, which is typical for avian viruses. Five of them (all H5N1), on the other hand, have this residue changed to Ser, which is mostly seen in human viruses. At the well-known position 627 of PB2, 5 sequences had gaps, 22 retained Glu (typical for avian virus), while the other 7 changed to Lys, which is typical for human virus. Among those 7 mutated sequences, 6 were from H5N1 human isolates (A/Hong Kong/483/1997, A/Hong Kong/485/1997, A/Vietnam/1194/2004, A/Vietnam/1203/2004, A/Vietnam/3062/2004, and A/Thailand/16/2004), and the other 1 was A/Netherlands/219/2003(H7N7), which was isolated from a fatal human case of pneumonia in the Netherlands (*32*).

**Table 2:** Summary of host-associated amino acid signature changes

Gene	Position	Residue*	H5N1	H9N2	H7N2	H7N7
PB2	199	A(19)	15	3		1
		S(5)	5			
	271	T(23)	20	2		1
		A(1)		1		
	627	E(22)	19	3		
		K(7)	6			1
PB1-F2	73	K(24)	17	2		5
		R(2)	2			
	79	R(24)	17	2		5
		Q(2)	2			
	82	L(21)	19	2		
		S(5)				5
PA	409	S(17)	12	3		2
		N(7)	7			
M2	20	S(34)	31	2	1	
		N(5)				5
NS2	70	S(26)	22	2		2
		G(1)		1		

*Top half displays an avian-specific residue with the count in parentheses and distribution among subtypes, and the bottom half represents a human-specific residue.

To understand how mutations had accumulated within a specific virus, we summarized the amino acid changes for 21 of these avian viruses that contained full or nearly full-length sequences for each segment (Table 3). We found that 19 of 21 strains contained >1 species-associated amino acid change, and 7 of them contained >2 substitutions; A/Netherlands/219/2003(H7N7) had the highest count for mutation accumulation (3 positions). Among these 52 species-associated signatures, the mutation combinations at positions PB2 199 and PA 409 were most commonly seen in H5N1 human isolates from Hong Kong in 1997.

**Table 3:** Twenty-one avian influenza A viral genomes isolated from humans and their mutations found at 12 host-associated positions within each strain*

Strain	Subtype	PB2			PB1-F2			PA	M2	NS2	Mutations
		199	271	627	73	79	82	409	20	70	
A/Hong Kong/156/1997	H5N1	**S**	T	E	K	R	L	**N**	S	S	2
A/Hong Kong/481/1997	H5N1	A	T	E	K	R	L	**N**	S	S	1
A/Hong Kong/482/1997	H5N1	**S**	T	E	K	R	L	**N**	S	S	2
A/Hong Kong/483/1997	H5N1	A	T	**K**	K	R	L	S	S	S	1
A/Hong Kong/485/1997	H5N1	A	T	**K**	#	#	#	S	S	S	1
A/Hong Kong/486/1997	H5N1	**S**	T	E	K	R	L	**N**	S	S	2
A/Hong Kong/532/1997	H5N1	A	T	E	K	R	L	**N**	S	S	1
A/Hong Kong/538/1997	H5N1	**S**	T	E	K	R	L	**N**	S	S	2
A/Hong Kong/542/1997	H5N1	A	T	E	K	R	L	**N**	S	S	1
A/Hong Kong/1997/1998	H5N1	**S**	T	E	K	R	L	S	S	S	1
A/Hong Kong/212/2003	H5N1	A	T	E	**R**	R	L	S	S	S	1
A/Hong Kong/213/2003	H5N1	A	T	E	**R**	R	L	S	S	S	1
A/Thailand/16/2004	H5N1	A	T	**K**	K	**Q**	L	S	S	S	2
A/Thailand/SP83/2004	H5N1	A	T	E	K	**Q**	L	S	S	S	1
A/Vietnam/1194/2004	H5N1	A	T	**K**	K	R	L	S	S	S	1
A/Vietnam/1203/2004	H5N1	A	T	**K**	K	R	L	S	S	S	1
A/Vietnam/3062/2004	H5N1	A	T	**K**	K	R	L	S	S	S	1
A/Netherlands/219/2003	H7N7	A	T	**K**	K	R	**S**	S	**N**	S	3
A/Guangzhou/333/1999	H9N2	A	**A**	E	#	#	#	S	S	**G**	2
A/Hong Kong/1073/1999	H9N2	A	T	E	K	R	L	S	R	S	0
A/Hong Kong/1074/1999	H9N2	A	T	E	K	R	L	S	S	S	0

*#indicates strains with PB1 RNA encoded into a truncated form of PB1-F2 of only 57 amino acids long. **Boldface** letters represent mutated (human-specific) residues; Roman (nonbold) letters are used for regular avian residue. Note that at position 20 of M2, A/Hong Kong/1073/99 had its residue changed from S to R, where R is still considered a mutation within avian species.

### RNA Segment 5

**Figure A2:**
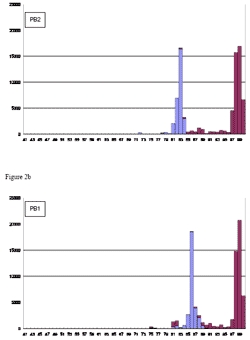
Histograms on comparing 306 human versus 95 avian influenza A viruses, based on nucleotide pairwise sequence identities. Vertical axis shows the count for pairs of sequences with specific percent identity (rounded to integer). Red bars represent frequencies for 'homo' pairs – sequences of the same host species (human to human, or avian to avian); blue bars represent frequencies for 'hetero' pairs – pairs that cross host species (human to avian, or avian to human). Adobe Acrobat PDF available at http://wwwnc.cdc.gov/eid/pdfs/06-0276-FA2.pdf (6 pages).

Our observation that NP contained the highest number (15 of 52) for species-associated amino acids suggested that NP might serve as a molecular target for differentiation between human and avian influenza A viruses. To indicate such host specificity, or the "genetic boundary" between these 2 viruses at the nucleotide level, we performed a pairwise sequence comparison for all 11 ORFs on our 401-genome primary dataset and produced histograms on their computed pairwise identities. In Figure A2, pairs with 2 sequences of the same host species (human to human, or avian to avian; termed homopairs) and pairs for sequences that cross host species (human to avian, or avian to human; termed heteropairs) are shown. HA and NA genes exhibited considerable sequence differences between strains, with identities as low as 47%. Also noted was a wide spectrum of percent identities (e.g., 55%–95% in the horizontal axis) containing few sequence pairs for these 2 genes. For both of these proteins, some strains from the same species can have identities as low as 50%. However, the ORF of another surface protein, M2 ion channel protein, is relatively conserved (>74% identity for viruses across species). The histograms for the polymerase genes (PB2, PB1, and PA), NP, and M1, on the other hand, are much less varied (mostly <20% variation). In particular, the NP gene was found to exhibit a fairly clear boundary between homopairs and heteropairs, at ≈86%.

## Discussion

The glutamic acid residue at PB2 627, which is commonly seen in avian viruses, restricts viral growth in humans and monkeys, but a change to lysine restores virus replication in mammalian cells (*33*). In this study we computed for every amino acid position (distributed in the 11 known influenza viral ORFs) an entropy value that represents how conserved an amino acid residue is at that given position. We found the entropy value –0.379 at 627 of PB2 and therefore used –0.4 as a threshold to discover other amino acid residues that might be potential determinants of host-cell tropism. Another 51 positions were found to be distinct or nearly distinct between human and avian viruses by this entropy threshold. Most of these (40 of 52) are located in viral ribonucleoproteins (RNPs) (PB2, PB1, PA, and NP), which are essential for viral replication. Taubenberger et al. reported 10 amino acid residues that distinguish human and avian influenza viral polymerases (*3*). Six of them were also identified in this study. The entropy values of the 4 missing ones were also found close to the preset threshold (–0.4). For example, PB2 567 showed a human entropy of –0.039 and avian entropy of –0.490, PB1 375 with human entropy –0.165 and avian entropy –0.693, and PA 100 with human entropy –0.061 and avian entropy –0.406. All 3 positions were eliminated earlier from the stage of analyzing the 401-genome primary dataset. The fourth position, PB2 702, although in the first-round list, marginally failed in the subsequent validation with human entropy –0.057 and avian entropy –0.404.

We proposed a computational approach capable of indicating species-associated signatures in studying human versus avian influenza viral genomes. Although we intended to analyze a comprehensive set of avian versus human influenza A viral genomes, the available sequences are predominated by H5N1 in avian viruses and H3N2 in human viruses. The short supply of sequences other than those 2 subtypes may inevitably cause a certain amount of bias in our results. At the completion of this study, we noticed a recent article by Obenauer et al., who had made 169 newly sequenced avian influenza viral genomes available to GenBank on January 26, 2006 (*34*); these were not included in our analysis. We checked on our 52 signature positions against these new genomes and found only 2 of them that showed an entropy value slightly over our threshold –0.4. These are PB1-F2 87 and HA 237, with entropy values of –0.522, and –0.692, respectively. The choice of entropy threshold would also affect the number of signatures found. Originally we chose –0.4 on the basis of the value –0.379, computed from PB2 627 by using 95 avian genomes. We noticed that this entropy value reduced to –0.299 at PB2 627 (see Table A4) at the later validation stage, when we found 197 E and 19 K from a total of 215 avian PB2 sequences. If we chose to use a more stringent entropy threshold of –0.3, our analysis still showed 46 of those 52 reported signatures; missing were positions 73, 79, and 82 from PB1-F2, 409 from PA, and 237 and 389 from HA.

In addition to the data limitations, this approach of looking for species-associated signatures by entropy is less useful for HA and NA genes. The genetic diversity that exists in either human or avian viruses for these 2 gene segments can markedly boost their respective entropy to more negative values, thus making it difficult to find residues conserved enough for identifying such signatures. We additionally performed the analysis on human H1, H2, and H3 versus avian HA (Figure A1). For NA we performed the analysis on human N1 and N2 versus avian NA. We compared 10 human H1, 3 human H2, and 293 human H3 with 95 avian HA sequences and found 13, 13, and 69 signatures (with entropy values for both human and avian within –0.4), respectively. This finding indicates that the human H1 and H2 strains are less distinct from avian strains (H5 dominant) than H3. For NA we found only 6 signatures, in comparison with 8 human N1 versus 95 avian (N1-dominant), and we found only 5 signatures when we compared 298 human N2 and 95 avian sequences. Entropy plots for these analyses can be seen in Figure A1.

**Figure A3:**
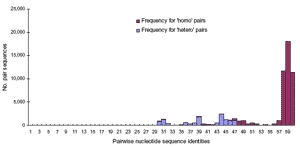
Histograms compare 43 avian allele A viruses and 306 human viruses (panels A and C), and 52 avian allele B viruses and 306 human viruses (panels B and D), based on their NS1 and NS2 genomic segments. Vertical axis shows the count for pairs of sequences with specific percent identity (rounded to integer). Red bars represent frequencies for 'homo' pairs – sequences of the same host species (human to human, or avian to avian); blue bars represent frequencies for 'hetero' pairs – pairs that cross host species (human to avian, or avian to human). Adobe Acrobat PDF available at http://wwwnc.cdc.gov/eid/pdfs/06-0276-FA3.pdf (5 pages).

Two genetic alleles (allele A and B) have been described for the NS gene in avian influenza A virus. We decomposed those 95 avian NS genes into 43 in allele A and 52 in allele B and compared their amino acid sequences with 306 human NS genes. For NS1, 6 signatures were found between human viruses and avian allele A viruses, and 35 signatures were found between human viruses and avian allele B viruses. For NS2, 3 signatures were found between human viruses and allele A viruses, and 6 signatures were found between human viruses and allele B viruses. These results suggest that avian allele B viruses are more distinct from human viruses than are allele A viruses. Entropy plots and histograms for these analyses can be seen in Figure A1 and Figure A3.

From the histograms, we found that some of the 11 genes vary greatly between human and avian viruses, while some others vary little. No boundaries were found between homopairs and heteropairs for HA, NA, and PB1 for human versus avian viruses. This finding seems reasonable because the 2 recent pandemic strains, the 1957 H2N2 and the 1968 H3N2, both originated from reassortment with avian influenza viruses (HA, NA, and PB1 gene segments were from avian influenza). On the other hand, because histograms of NP, followed by PA and PB2, may be used to distinguish human influenza viruses from avian influenza viruses, perhaps some biologic constraints against the occurrence of reassortment exist for these 3 genes. Both the M and NS genes are less differentiable between these 2 types of influenza A viruses.

NP not only displays a clear boundary between human and avian viruses from histogram analysis but also contains more species-associated amino acid signatures (15 of 52) than other ORFs. In addition to NP, polymerase proteins PB2, PB1, and PA also contain abundant species-associated signatures. Most signatures in these viral RNPs are located on the functional domains related to RNP-RNP interactions that are necessary to form replicase/transcriptase complex (3P and NP), which suggests that specific combinations of polymerase complex and NP would allow an influenza virus to replicate itself efficiently (Table 1). In addition to RNA-interacting domains, many species-associated amino acid signatures of 3P and NP are located in regions related to nuclear localization signals. Influenza viral replication is highly dependent on nuclear function (*35*), making it worthwhile to further examine the roles of those amino acid signatures on nuclear localization of viral RNP in avian versus human cells. We also noticed that several amino acid signatures in NP are located in the regions that interact with cellular proteins, such as splicing factor (BAT1/UAP56) or MxA, which plays a certain role in cellular antiviral mechanisms. What species-specific host factors may affect influenza viral replication rates is not clear. Biologic experiments are required for further understanding the roles of those amino acid residues and related functional domains in the mechanism of interspecies infection.

PB1-F2 is a novel influenza viral protein translated from alternative initiation of PB1 gene. PB1-F2 of PR8 (H1N1) has been shown to target mitochondria and then trigger host cell apoptosis (*36*). Our previous research has found that several strains contain truncated PB1-F2 (*37*). In this study, 379 of 401 PB1 sequences (in the primary dataset) contained PB1-F2 >87 and <90 aa. For the other 22 sequences, 2 H3N2 strains missed a start codon, 3 H3N2 had the translation stopped at 11 aa, 1 H9N2 stopped at 8 aa, 5 H1N1 stopped at 57 aa, and 3 H9N2 and 7 H3N2 stopped at 79 aa. One H5N1 contained extra residues; its PB1-F2 was 101 aa. We also noted 5 species-associated signatures on PB1-F2; all of them are within the C-terminal domain, which is important for mitochondria targeting (*15**,**16*). Further investigation of the mitochondria localization of those PB1-F2 variants and their abilities for triggering apoptosis in cells derived from different species is warranted.

How many mutations would make an avian virus capable of infecting humans efficiently, or how many mutations would render an influenza virus a pandemic strain, is difficult to predict. We have examined sequences from the 1918 strain, which is the only pandemic influenza virus that could be entirely derived from avian strains. Of the 52 species-associated positions, 16 have residues typical for human strains; the others remained as avian signatures. The result supports the hypothesis that the 1918 pandemic virus is more closely related to the avian influenza A virus than are other human influenza viruses (*2*). From the 21 avian viruses isolated from humans in this study, we found 19 (90.5%) that contain >1 change at the species-associated sites. Upon examining signature changes from similarly sized sets of randomly selected human viruses, randomly selected avian viruses, and randomly selected viruses (avian plus human), we found 29.4%, 71.4%, and 47.1%, respectively, contain species-associated mutations. Although predicting the emergence of a pandemic strain is difficult, close monitoring of how those species-associated signature positions have changed from bird-specific to human-specific signatures may provide a measurement for the prediction of such events.

## Appendix

### Supporting Materials and Methods

In the main text we have mentioned an entropy value was defined at an aligned amino acid position according to the formula ΣPi*log(Pi), where *i* is the observed probability for each of the 20 amino acids. An entropy value defined like this is at most zero when all amino acids at this position conserve to the same residue, while a more negative value indicates that the residues are more divergent for containing more residue types. Although BioEdit also includes a module with similar formula in computing entropy values for aligned sequences, we chose to develop our own software for more streamlined data manipulation and subsequent analysis and interpretation.

To reveal the host-associated amino acid signatures, we have retrieved full genome sequences (as of August 22, 2005) from the genome browser at Influenza Sequence Database. Strains containing all eight RNA segments and for each segment a minimum 90% long of the coding sequence based on PR8 were included, which serve as the primary dataset for full genome scanning. Altogether, we have 95 avian influenza genomes (including 60 H5N1, 8 H6N1, 6 H6N2, 1 H7N1, 1 H7N3, 2 H7N7, 17 H9N2) and 306 human influenza genomes (8 H1N1, 2 H1N2, 3 H2N2 and 293 H3N2), the latter include 11 complete genomes of Taiwanese strains from 1996 to 2004 (newly sequenced data from this study). See Supporting Table 1 for a complete listing of accessions for these 401 genomes. Coding sequence alignments for each genomic segment were compiled: PB2, 759 aa; PB1, 757 aa; PB1-F2, 90 aa; PA, 716 aa; HA, 591 aa; NP, 498 aa; NA, 480 aa; M1, 252 aa; M2, 97 aa; NS1, 230 aa; and NS2, 121 aa.

Human-isolated avian influenza viruses from human flu were separately retrieved from NCBI as well as from ISD. Altogether we have 417 accessions from 60 avian flu strains (48 H5N1, 6 H9N2, 5 H7N7 and 1 H7N2), in which 21 strains (17 H5N1, 3 H9N2 and 1 H7N7) contain sequences (full or nearly full-length) from all 8 genomic RNAs. See Table A2 for a complete listing of these accessions.

For validating the obtained signatures from analyzing the mentioned 401-genome primary dataset, we have firstly retrieved 14,057 human or avian influenza A protein sequences from NCBI's Influenza Virus Resources (as of January 17, 2006), including 5,468 avian and 8,589 human sequences (786 H1N1 sequences and 7,097 H3N2 sequences among the others). At the stage of revising this manuscript, we have included more H1N1sequences (2,514 in total, as of April 20, 2006) for validation to relieve the limitation that may be caused by the unbalanced sequence counts between H1N1 (786 sequences) and H3N2 (7,097 sequences) previously used, thus making the results more robust. Altogether we have used 15,785 influenza protein sequences for confirmatory analysis.

## Appendix

**Table A1:** Listing of 401 genomes used in this study. All accessions are according to GenBank, except for A/Puerto Rico/8/34(H1N1), which are from Influenza Sequence Database (ISD). Full table available at www.cdc.gov/eid-static/spreadsheets/06-0276-TA1.xlsx.

Strain	Subtype	Host	PB2	PB1	PA	HA	NP	NA	M	NS
A/BAR-HEADED GOOSE/QINGHAI/5/05	H5N1	Avian	DQ095757	DQ095737	DQ095717	DQ095617	DQ095677	DQ095657	DQ095637	DQ095697
A/BAR-HEADED GOOSE/QINGHAI/59/05	H5N1	Avian	DQ095752	DQ095732	DQ095712	DQ095612	DQ095672	DQ095652	DQ095632	DQ095692
A/BAR-HEADED GOOSE/QINGHAI/60/05	H5N1	Avian	DQ095755	DQ095735	DQ095715	DQ095615	DQ095675	DQ095655	DQ095635	DQ095695
A/BAR-HEADED GOOSE/QINGHAI/61/05	H5N1	Avian	DQ095758	DQ095738	DQ095718	DQ095618	DQ095678	DQ095658	DQ095638	DQ095698
A/BAR-HEADED GOOSE/QINGHAI/62/05	H5N1	Avian	DQ095760	DQ095740	DQ095720	DQ095620	DQ095680	DQ095660	DQ095640	DQ095700
A/BAR-HEADED GOOSE/QINGHAI/65/05	H5N1	Avian	DQ095762	DQ095742	DQ095722	DQ095622	DQ095682	DQ095662	DQ095642	DQ095702
A/BAR-HEADED GOOSE/QINGHAI/67/05	H5N1	Avian	DQ095763	DQ095743	DQ095723	DQ095623	DQ095683	DQ095663	DQ095643	DQ095703
A/BAR-HEADED GOOSE/QINGHAI/68/05	H5N1	Avian	DQ095753	DQ095733	DQ095713	DQ095613	DQ095673	DQ095653	DQ095633	DQ095693
A/BAR-HEADED GOOSE/QINGHAI/75/05	H5N1	Avian	DQ095759	DQ095739	DQ095719	DQ095619	DQ095679	DQ095659	DQ095639	DQ095699
A/BIRD/THAILAND/3.1/2004	H5N1	Avian	AY651715	AY651661	AY651607	AY651330	AY651495	AY651441	AY651384	AY651550
A/BROWN-HEADED GULL/QINGHAI/3/05	H5N1	Avian	DQ095756	DQ095736	DQ095716	DQ095616	DQ095676	DQ095656	DQ095636	DQ095696
A/CHICKEN/BEIJING/1/94	H9N2	Avian	AF156438	AF156423	AF156452	AF156380	AF156409	AF156398	AF156466	AF156480
A/CHICKEN/BEIJING/8/98	H9N2	Avian	AF508649	AF508627	AF508671	AF508562	AF508605	AF508583	AF508693	AF508714
A/CHICKEN/BRITISH COLUMBIA/04	H7N3	Avian	AY616766	AY616765	AY616764	AY611524	AY611527	AY611526	AY611525	AY611528
A/CHICKEN/CALIFORNIA/139/01	H6N2	Avian	AF457705	AF457706	AF457707	AF457713	AF474070	AF457711	AF457712	AF457708
A/CHICKEN/CALIFORNIA/431/00	H6N2	Avian	AF457697	AF457698	AF457699	AF457704	AF457701	AF457702	AF457703	AF457700
A/CHICKEN/CALIFORNIA/465/00	H6N2	Avian	AF457689	AF457690	AF457691	AF457696	AF457693	AF457694	AF457695	AF457692
A/CHICKEN/CALIFORNIA/6643/01	H6N2	Avian	AF457681	AF457682	AF457683	AF457688	AF457685	AF457686	AF457687	AF457684
A/CHICKEN/CALIFORNIA/905/01	H6N2	Avian	AF457672	AF457673	AF457674	AF457679	AF457676	AF457677	AF457678	AF457675
A/CHICKEN/GERMANY/R28/03	H7N7	Avian	AJ620347	AJ620348	AJ619677	AJ620350	AJ620352	AJ620349	AJ619676	AJ619678
A/CHICKEN/GUANGDONG/10/00	H9N2	Avian	AF508650	AF508628	AF508672	AF508563	AF508606	AF508584	AF508694	AF508715
A/CHICKEN/GUANGDONG/11/97	H9N2	Avian	AF508651	AF508629	AF508673	AF508564	AF508607	AF508585	AF508695	AF508716
A/CHICKEN/GUANGDONG/174/04	H5N1	Avian	AY609309	AY609310	AY609311	AY609312	AY609313	AY609314	AY609315	AY609316
A/CHICKEN/GUANGDONG/178/04	H5N1	Avian	AY737293	AY737294	AY737295	AY737296	AY737297	AY737299	AY737298	AY737300
A/CHICKEN/GUANGDONG/191/04	H5N1	Avian	AY737286	AY737287	AY737288	AY737289	AY737290	AY737291	AY737292	AY737285
A/CHICKEN/HONG KONG/220/97	H5N1	Avian	AF046086	AF046085	AF046087	AF046080	AF046084	AF046081	AF046082	AF046083
A/CHICKEN/HONG KONG/728/97	H5N1	Avian	AF098579	AF098592	AF098606	AF046099	AF098618	AF098548	AF098562	AF098571
A/CHICKEN/HONG KONG/739/94	H9N2	Avian	AF156436	AF156422	AF156450	AF156379	AF156408	AF156397	AF156464	AF156478
A/CHICKEN/HONG KONG/FY150/01	H5N1	Avian	AY221587	AY221578	AY221569	AY221524	AF509120	AF509095	AF509043	AY221560
A/CHICKEN/HONG KONG/NT873.3/01	H5N1	Avian	AY221585	AY221576	AY221567	AY221522	AY221549	AY221540	AY221531	AY221558
A/CHICKEN/HONG KONG/YU562/01	H5N1	Avian	AY221592	AY221583	AY221574	AY221529	AF509118	AY221547	AF509041	AF509067
A/CHICKEN/HONG KONG/YU822.2/01	H5N1	Avian	AY221591	AY221582	AY221573	AY221528	AY221555	AY221546	AY221537	AY221564

**Table A2:** Listing of 60 human-isolated avian influenza viruses used in this study, with the first 29 strains contain at least one accession per genomic segment. All accessions here are according to GenBank, except those ones begin with 'ISD', which are from Influenza Sequence Database (ISD). Cells with 'n/a' in PB1-F2 column indicate that the PB1 RNA sequence did not contain the PB1-F2 ORF, while 'truncated' represent early-terminated PB1-F2 with a length less than 87-aa. Exclusion of those PB1-F2 leaves us 21 genomes for inspecting the species-associated mutations as described in the text.

Strain Name	PB2	PB1	PB1-F2	PA	HA	NP	NA	M1	M2	NS1	NS2
A/HongKong/156/97(H5N1)	AF036363	AF036362	AF036362	AF084267	AF028709	AF028710	AF036357	AF036358	AF036358	AF036360	AF036360
A/HongKong/481/97(H5N1)	AF115290	AF258818	AF258818	AF115294	AF046096	AJ289873	AF084271	AF115286	AF115286	AF115288	AF115288
A/HongKong/482/97(H5N1)	AF258838	AF258819	AF084264	AF084268	AF046098	AF255745	AF084272	AF084282	AF084282	AF084285	AF084285
A/HongKong/483/97(H5N1)	AF258839	AF258820	AF084265	AF084269	AF046097	AF084277	AF084273	AF255367	AF255367	AF084286	AF084286
A/HongKong/485/97(H5N1)	AF084263	AF084266	truncated	AF084270	AF102681	AF084278	AF084274	AF084284	AF084284	AF084287	AF084287
A/HongKong/486/97(H5N1)	AF115291	AF115293	AF115293	AF115295	AF102671	AF115285	AF084275	AF255368	AF255368	AF256181	AF256181
A/HongKong/488/97(H5N1)	AF258848	AF258829	n/a	AF257204	AF102672	AF255756	AF102657	AF255377	AF255378	AF256190	AF256190
A/HongKong/491/97(H5N1)	AF258849	AF258830	n/a	AF257205	AF102677	AF255758	AF102665	AF255379	AF255380	AF256191	AF256191
A/HongKong/503/97(H5N1)	AF258850	AF258831	n/a	AF257206	AF102679	AF255760	AF102666	AF255381	AF255381	AF256192	AF256192
A/HongKong/507/97(H5N1)	AF258851	AF258832	n/a	AF257207	AF102675	AF255762	AF102659	AF255382	AF255382	AF256193	AF256193
A/HongKong/514/97(H5N1)	AF258852	AF258833	n/a	AF257208	AF102682	AF255764	AF102669	AF255383	AF255383	AF256184	AF256184
A/HongKong/516/97(H5N1)	AF258853	AF258834	n/a	AF257209	AF102673	AF255766	AF102660	AF255384	AF255384	AF256194	AF256194
A/HongKong/532/97(H5N1)	AF258843	AF258824	AF258824	AF257199	AF102680	AF255750	AF102667	AF255371	AF255371	AF256185	AF256185
A/HongKong/538/97(H5N1)	AF258844	AF258825	AF258825	AF257200	AF102674	AF255751	AF102662	AF255372	AF255372	AF256186	AF256186
A/HongKong/542/97(H5N1)	AF258845	AF258826	AF258826	AF257201	AF102678	AF255752	AF102670	AF255373	AF255373	AF256187	AF256187
A/HongKong/97/98(H5N1)	AF258846	AF258827	AF258827	AF257202	AF102676	AF255753	AF102661	AF255374	AF255374	AF256188	AF256188
A/HongKong/212/03(H5N1)	AY576380	AY576392	AY576392	AY576404	AY575869	AY575905	AY575881	AY575893	AY575893	AY576368	AY576368
A/HongKong/213/2003(H5N1)	AY576381	AB212052	AY576393	AB212053	AB212054	AB212055	AB212056	AB212057	AB212057	AY576369	AY576369
A/Thailand/16/2004(H5N1)	ISDN40383	ISDN40859	ISDN40859	ISDN40940	ISDN40341	ISDN40086	ISDN48790	ISDN45755	ISDN45755	ISDN40040	ISDN40040
A/Thailand/SP83/2004(H5N1)	ISDN49457	ISDN40931	ISDN40931	ISDN121933	ISDN40917	ISDN41067	ISDN48792	ISDN111182	ISDN111182	ISDN41028	ISDN41028
A/Vietnam/1194/2004(H5N1)	AY651718	AY651664	AY651664	AY651610	AY651333	AY651498	ISDN38703	ISDN39957	ISDN39957	AY651552	AY651552
A/Vietnam/1196/04(H5N1)	AY526752	AY526751	AY526751	AY526750	AY526745	AY526749	AY526746	AY526748	AY526748	AY526747	AY526747
A/Vietnam/1203/2004(H5N1)	AY651719	AY818129	AY651665	AY818132	ISDN38687	AY818138	AY651447	AY651388	AY651388	AY651553	AY651553
A/Vietnam/3046/2004(H5N1)	AY651720	AY651666	AY651666	AY651613	AY651335	AY651500	AY651446	AY651389	AY651389	AY651554	AY651554
A/Vietnam/3062/2004(H5N1)	AY651721	AY651667	AY651667	AY651612	AY651336	AY651501	AY651448	AY651390	AY651390	AY651555	AY651555
A/Netherlands/219/03(H7N7)	AAR04358	AAR05983	AY340083	AAR04363	AAR02640	AAR04370	AAR11367	AAR11371	AY340089	AAR04367	AY342422
A/Guangzhou/333/99(H9N2)	AY043030	AY043029	truncated	AY043028	AY043019	AY043026	AY043024	AY043025	AY043025	AY043027	AY043027
A/HongKong/1073/99(H9N2)	AF258835	AF258816	AF258816	AF257191	AJ404626	AJ289871	AJ404629	AF255363	AF255363	AJ278649	AJ278649
A/HongKong/1074/99(H9N2)	AF258836	AF258817	AF258817	AF257192	AJ404627	AJ289872	AJ404628	AF255364	AF255364	AF256177	AF256177
A/England/268/96(H7N7)					AF028020						
A/Shantou/239/98(H9N2)					AY043015		AY043021				
A/Shaoguan/408/98(H9N2)					AY043017		AY043022				
A/Shaoguan/447/98(H9N2)					AY043018		AY043023				
A/unknown/149717-12/2002(H7N2)								DQ107480	DQ107480		
A/Netherlands/124/03(H7N7)	AAR04355	AAR05980	AY340080	AAR04360			AAR11364	AAR11368	AY340086		
A/Netherlands/126/03(H7N7)	AAR04356	AAR05981	AY340081	AAR04361			AAR11363	AAR11369	AY340087		
A/Netherlands/127/03(H7N7)	AAR04357	AAR05982	AY340082	AAR04362	AAR02636			AAR11370	AY340088	AAR04366	AY342421
A/Netherlands/33/03(H7N7)		AAR05984	AY340084	AAR04364	AAR02638	AAR04371	AAR11366	AAR11372	AY340090	AAR04368	AY342423
A/Hanoi/03/2004(H5N1)					AJ715872	AJ715873					
A/Hatay/2004(H5N1)					AJ867074	AJ867076	AJ867075	AM040045	AM040045	AM040046	AM040046
A/Prachinburi/6231/2004(H5N1)					ISDN110940		ISDN110939				
A/Thailand/1-KAN-1/2004(H5N1)					AY555150		AY555151				
A/Thailand/2-SP-33/2004(H5N1)					AY555153		AY555152				
A/Thailand/Chaiyaphum/622/2004(H5N1)					ISDN49460		ISDN48793	ISDN111184	ISDN111184		
A/Thailand/EKA2NF/2004(H5N1)							AY535029				
A/Thailand/Kamphaengphet-Nontaburi/04(H5N1)					AY786078		AY786079				
A/Thailand/Kan353/2004(H5N1)					ISDN40918		ISDN48791	ISDN111183	ISDN111183		
A/Thailand/Prachinburi/6231/2004(H5N1)								ISDN111185	ISDN111185		
A/Thailand/LFPN-2004/2004(H5N1)					AY679514		AY679513				
A/Vietnam/1194/2004(H5N1)					ISDN38686		AY651445	AY651387	AY651387		
A/Vietnam/1204/2004(H5N1)	ISDN40380	ISDN40843	ISDN40843	ISDN121932	ISDN38688					ISDN40017	ISDN40017
A/Vietnam/3212/2004(H5N1)					ISDN40278						
A/Vietnam/DN-33/2004(H5N1)					AY720950		AY720948			AY720949	AY720949
A/Vietnam/JP178/2004(H5N1)					ISDN69608		ISDN69610				
A/Vietnam/HN/2004(H5N1)	AY720954	AY720955	n/a	AY720952		AY720953		AY720951	AY720951		
A/Cambodia/JP52a/2005(H5N1)					ISDN121986		ISDN122818				
A/Hanoi/30408/2005(H5N1)					ISDN129400						
A/Vietnam/HN30408/2005(H5N1)					ISDN119678		ISDN119679				
A/Vietnam/JP14/2005(H5N1)					ISDN117778		ISDN117783				
A/Vietnam/JP4207/2005(H5N1)					ISDN117777		ISDN117782				
A/Vietnam/JPHN30321/2005(H5N1)					ISDN118371						

**Table A3:** Genome-scanning for 228 amino acid 'signatures' (those ones shown in bold face, either 'Distinct' or 'Nearly Distinct') out of 4,591 aligned amino acid positions. Con: consensus residue; Ent: entropy value; Same: all avian and human strains conserve to the same residue; Nearly Identical: both avian and human strains have entropy values less negative than -0.4, and have the same residue; Distinct: both avian and human strains contain zero entropy yet conserve to the different residue; Nearly Distinct: both avian and human strains contain an entropy value less negative than -0.4, and conserve to different residue. The last column shows positions based on PR8. Only HA and NA have different numberings comparing with the first column, due to excessive shifting of amino residues from HA and NA genetic diversity. Full table available at www.cdc.gov/eid-static/spreadsheets/06-0276-TA3.xlsx.

Scanning for amino acid 'signatures' for influenza A virus PB2 protein								
Pos	Avian			Human			Comments	PR8
	Con	Ent	Residues	Con	Ent	Residues		
1	M	-0.500	M(76),-(19),	M	-0.055	M(303),-(3),		
2	E	-0.293	E(88),K(1),-(6),	E	-0.061	E(303),V(1),-(2),	Nearly Identical	
3	R	-0.175	R(91),-(4),	R	-0.022	R(305),T(1),	Nearly Identical	
4	I	-0.140	I(92),-(3),	I	-0.022	I(305),L(1),	Nearly Identical	
5	K	-0.140	K(92),-(3),	K	-0.039	R(2),K(304),	Nearly Identical	
6	E	-0.140	E(92),-(3),	E	0.000	E(306),	Nearly Identical	
7	L	-0.160	L(92),F(1),-(2),	L	0.000	L(306),	Nearly Identical	
8	R	-0.160	R(92),W(1),-(2),	R	0.000	R(306),	Nearly Identical	
9	D	-0.521	N(1),D(83),E(7),Y(2),-(2),	N	-0.619	N(227),D(1),S(2),T(76),		
10	L	-0.160	I(1),L(92),-(2),	L	-0.022	I(1),L(305),	Nearly Identical	
11	M	-0.117	I(1),M(93),-(1),	M	0.000	M(306),	Nearly Identical	
12	S	0.000	S(95),	S	-0.022	L(1),S(305),	Nearly Identical	
13	Q	0.000	Q(95),	Q	0.000	Q(306),	Same	
14	S	0.000	S(95),	S	-0.022	F(1),S(305),	Nearly Identical	
15	R	0.000	R(95),	R	0.000	R(306),	Same	
16	T	-0.058	S(1),T(94),	T	0.000	T(306),	Nearly Identical	
17	R	0.000	R(95),	R	0.000	R(306),	Same	
18	E	0.000	E(95),	E	0.000	E(306),	Same	
19	I	0.000	I(95),	I	0.000	I(306),	Same	
20	L	0.000	L(95),	L	-0.022	L(305),V(1),	Nearly Identical	
21	T	0.000	T(95),	T	0.000	T(306),	Same	
22	K	0.000	K(95),	K	-0.022	N(1),K(305),	Nearly Identical	
23	T	0.000	T(95),	T	-0.022	P(1),T(305),	Nearly Identical	
24	T	0.000	T(95),	T	0.000	T(306),	Same	
25	V	0.000	V(95),	V	0.000	V(306),	Same	

**Table A4:** Amino acid 'signatures' validation. Only positions with both newly computed entropy values less or equal to -0.400 are considered 'validated'. This reduces 228 signatures to a count of 52 (the ones shown in bold face) as reported in the manuscript. Cnt: total number of avian or human residues at this position; Ent: computed entropy value; PR8: position numbering based on PR8 (only HA and NA have different numbering here). Full table available at www.cdc.gov/eid-static/spreadsheets/06-0276-TA4.xlsx.

Gene	Pos	Avian influenza viruses		Human influenza viruses				Validated?	PR8
		Cnt	Ent	Residues	Cnt	Ent	Residues		
PB2	44	215	-0.144	A(208),S(7),	843	-0.081	A(10),L(2),S(831),	Yes	
	67	215	-0.174	I(206),V(9),	843	-0.538	I(193),V(650),		
	81	215	-0.196	A(2),I(7),T(206),	843	-0.537	I(2),L(4),M(686),T(4),V(147),		
	82	215	-0.164	R(1),N(209),K(1),S(2),T(2),	843	-0.608	N(180),C(14),S(648),X(1),		
	120	215	0.000	E(215),	843	-0.575	N(1),D(628),E(214),		
	199	215	-0.110	A(210),S(5),	845	-0.024	A(3),S(842),	Yes	
	227	215	0.000	V(215),	845	-0.697	I(586),M(19),V(240),		
	271	215	-0.133	A(3),I(1),M(1),T(210),	843	-0.051	A(836),S(1),T(6),	Yes	
	382	215	-0.110	I(210),V(5),	842	-0.527	I(185),V(657),		
	453	215	-0.164	Q(2),H(1),L(2),P(209),S(1),	842	-0.497	R(1),H(691),L(1),P(147),S(2),		
	456	215	-0.219	N(205),D(6),S(4),	842	-0.623	N(247),D(1),C(1),S(593),		
	461	215	-0.159	I(207),V(8),	842	-0.638	I(283),V(559),		
	463	215	-0.247	I(203),L(1),M(1),V(10),	842	-0.529	I(181),M(1),V(660),		
	475	215	-0.030	L(214),M(1),	842	-0.024	L(3),M(839),	Yes	
	478	215	-0.484	I(30),L(1),M(2),V(182),	842	-0.541	I(656),L(2),V(184),		
	526	215	-0.053	R(2),K(213),	841	-0.577	R(619),K(222),		
	559	215	-0.255	I(5),M(2),T(204),V(4),	841	-0.694	A(547),N(1),I(2),T(287),V(4),		
	588	215	-0.254	A(203),T(6),V(6),	841	-0.050	A(2),I(835),V(3),X(1),	Yes	
	613	215	-0.073	A(3),V(212),	841	-0.157	A(8),I(16),T(816),V(1),	Yes	
	627	215	-0.299	E(196),K(19),	841	-0.026	R(2),E(1),K(838),	Yes	
